# Antibacterial and Bioregenerative Nanomaterials in Oral Health: From Material Design to Clinical Translation and Technological Trends

**DOI:** 10.3390/jfb17020087

**Published:** 2026-02-10

**Authors:** Dana Emanuela Pitic (Cot), Aniela-Roxana Nodiți-Cuc, Cristina Ioana Talpos-Niculescu, Diana Marian, Ramona Amina Popovici, Andreea Mihaela Kis, Laria-Maria Trusculescu, Adina Feher, Ioana Elena Lile

**Affiliations:** 1 Management and Communication in Dental Medicine Department I, Faculty of Dental Medicine, Victor Babes University of Medicine and Pharmacy, Piața Eftimie Murgu 2, 300041 Timisoara, Romania; dana.pitic@umft.ro (D.E.P.); ioana.talpos-niculescu@umft.ro (C.I.T.-N.); kis.andreea@umft.ro (A.M.K.); laria.trusculescu@umft.ro (L.-M.T.); adina.feher@umft.ro (A.F.); 2 Department of Surgical Oncology, ‘Carol Davila’ University of Medicine and Pharmacy, 020021 Bucharest, Romania; aniela.noditi@umfcd.ro; 3 Department of Dentistry, Faculty of Dentistry, “Vasile Goldiș” Western University of Arad, 94-96 Revolutiei Blvd., 310025 Arad, Romania; lile.ioana@uvvg.ro

**Keywords:** antibacterial nanomaterials, nanohydroxyapatite, bioglass, bioactive nanocomposites, regenerative dental materials, restorative dentistry, 3D printing, artificial intelligence

## Abstract

Context: The increasing incidence of secondary caries and the failure of restorations have intensified research into dental restorative materials capable of actively interacting with the oral environment. In this context, antibacterial and bioregenerative nanomaterials have attracted growing scientific interest due to their potential to inhibit biofilm formation while simultaneously supporting mineral repair processes. Objective: This narrative review analyzes recent developments in nanostructured materials for restorative dentistry and oral health applications, with particular emphasis on antibacterial agents, bioactive systems, and emerging dual-function approaches that integrate multiple biological functions into restorative materials. Scope of the Review: The analyzed literature indicates that metallic nanoparticles, cationic monomers, and natural nanopolymers can reduce bacterial adhesion and metabolic activity under experimental conditions. In parallel, bioactive nanomaterials such as nanohydroxyapatite, bioactive glass, and calcium phosphate-based systems have demonstrated the ability to release remineralizing ions and to promote mineral deposition at the tooth–material interface. Dual-function hybrid materials aim to combine these antibacterial and bioregenerative effects within a single restorative system. Interpretative Perspective: Despite these advances, most available evidence derives from in vitro and preclinical studies, with significant heterogeneity across experimental models, evaluation methods, and outcome variables. This variability limits direct comparisons between studies and necessitates a cautious interpretation of claims regarding long-term antibacterial efficacy, functional tissue regeneration, and routine clinical applicability. Conclusions: Antibacterial and bioregenerative nanomaterials represent a relevant and continuously evolving research direction in restorative dentistry. Their successful clinical translation will depend on establishing standardized testing protocols, conducting comprehensive safety assessments, and generating clinically relevant evidence supporting long-term efficacy and biological compatibility. Their successful clinical translation will depend on establishing standardized testing protocols, conducting comprehensive safety assessments, and generating clinically relevant evidence supporting long-term efficacy and biological compatibility.

## 1. Introduction

Dental caries represents the most prevalent non-communicable disease (NCD), which affects millions of people across the globe. The development of this condition results from multiple factors, including biofilm development and interactions among oral microbial communities, individual behaviors or dietary choices, and environmental factors [1,2]. Current ecological models describe caries development through disturbances in biofilm caused by repeated exposure to fermentable carbohydrates in the oral cavity. At the same time, the local pH environment shifts between acidic and basic states [3].

The public health community classifies caries as a non-communicable disease because its development stems from behavioral choices and it persists as a chronic condition [4,5]. The FDI White Paper (2016), along with other essential oral health documents, indicates that social inequality determines worldwide caries prevalence, as prevention programs should teach individuals to prevent caries development through risk factor control [6].

The ideal goal of restorative dentistry is not merely to fill a cavity, but to achieve a seamless biological integration of the restorative material, ensuring the protection of the pulp-dentin complex and the long-term functional stability of the tooth. The development of restorative materials during the previous several decades has led to improved clinical outcomes. The current resin composites demonstrate better properties, including enhanced bonding strength and mechanical performance, as well as an improved esthetics [7,8]. However, the oral environment needs bioactive therapeutic solutions that prevent biofilm development and support tooth mineralization, replacing conventional dental restoratives in modern dental practice [9,10]. These requirements employ bio-active materials, including bioglass, nanostructured hydroxyapatite, and ion-releasing resin composites, to help restore mineral content and promote tissue healing [11,12,13,14]. It is crucial to precisely define the term bioregeneration in this context, distinguishing between remineralization (the mineral repair process at the tooth–material interface), bioactivity (the ability of a material to form a layer of apatite on its surface when in contact with physiological fluids), and true regeneration (complete tissue regeneration which brings cells to the site and leads to dentin bridge development and pulp recovery).

The primary aim of restorative dentistry is not limited to the mechanical replacement of lost tooth structure, but rather to restore the biological, functional, and structural integrity of the tooth while maintaining long-term compatibility with the oral environment. An ideal restorative treatment should reestablish masticatory function, marginal sealing, and esthetics, while simultaneously minimizing the risk of secondary caries, pulpal inflammation, and material degradation over time. Accordingly, ideal restorative biomaterials are expected to meet different functional requirements depending on the clinical indication. For direct restorations, materials should combine adequate mechanical strength with antibacterial properties and stable adhesion to enamel and dentin to limit biofilm accumulation and marginal leakage. In indirect restorations, long-term structural stability, resistance to wear, and interfacial integrity become critical. For deep restorations and vital pulp therapies, bioactive and bioregenerative properties—such as ion release, pH buffering, and stimulation of reparative dentin formation—are particularly desirable to support tissue healing and preserve pulp vitality.

These evolving clinical objectives have driven the development of functional and dual-function restorative materials that aim to actively interact with the oral environment rather than act as passive fillers.

Also, the advancement of complex restorative materials depends on nanotechnology, which serves as their fundamental technological base. Scientists use nanoscale particles to develop antibacterial and remineralizing materials because their expanded surface area, elevated reactivity, and functionalization capabilities exceed those of conventional systems [15,16]. The scientific community actively studies metallic nanoparticles (Ag, ZnO, TiO_2_), quaternary ammonium monomers (QAM), and calcium-phosphate-based nanocomposites (NACP) because they influence biofilm formation and prevent further tooth decay. Multiple studies have shown that metallic nanoparticles exhibit antibacterial properties, with higher concentrations effectively inactivating bacteria, but researchers need to find the right balance between tissue safety and treatment success [17,18,19,20]. Dual-function nanomaterials represent a new research field, as they combine antibacterial properties with tissue-regeneration capabilities [21,22]. In the context of oral health applications, functional materials are defined as restorative systems that actively interact with the biological environment by expressing a single dominant biological function beyond passive structural replacement. In contrast, dual-function materials are integrated nanomaterial systems specifically designed to concurrently express two distinct and complementary biological functions—namely, antibacterial activity and bioregenerative (remineralizing or regenerative) capacity—within the same material platform. Notably, dual-function systems are characterized not merely by the coexistence of multiple components, but by the simultaneous and functionally relevant operation of both mechanisms under clinically relevant conditions. The nanocomposites composed of NACP and QAM demonstrate potential for creating sophisticated “smart” restorative materials because they inhibit *Streptococcus mutans* proliferation through adaptive release of Ca^2+^/PO_4_^3−^ ions [23,24,25].

The field of nanomaterials continues to develop through technological advancements, 3D printing, and AI-based design, but most of the currently available evidence is derived from in vitro and preclinical studies [26,27,28,29]. While experimental studies have demonstrated ion release, dentin interaction, and interfacial bioactivity of modern bioceramic materials, clinical adoption remains inconsistent [7,8]. Although, analyses of dentin–sealer interfaces confirm the potential biological advantages of bioactive sealers, yet survey data from dental practices reveal variability in material selection and limited standardization in clinical use. These findings highlight the persistent translational gap between advances in materials science and everyday dental practice [30,31].

This narrative review synthesizes representative research on nanomaterials in dental restorations published between 2015 and 2025, with emphasis on antibacterial and remineralizing properties, material characteristics, and emerging clinical considerations. It evaluates experimental and preclinical findings, identifying approaches with significant potential and those requiring further validation through rigorous clinical studies. Current literature encompasses multiple reviews on antibacterial nanomaterials and bioactive regenerative materials. However, few narrative syntheses address these approaches collectively within the context of multifunctional restorative strategies.

## 2. Materials and Methods

A narrative synthesis was conducted to analyze current advancements in antibacterial and bioregenerative nanomaterials for restorative dentistry. The literature search was completed on 15 November 2025. A harmonized search string was utilized across three major databases (PubMed, Web of Science, and Scopus): (“antibacterial nanomaterials” OR “bioregenerative nanomaterials” OR “bioactive restorative materials”) AND (“restorative dentistry” OR “dental restoration”).

The selection process followed a structured workflow:

1. Identification: The initial search generated a total of 485 records.
2. Deduplication: After removing duplicates, 312 unique studies remained for evaluation.
3. Screening: Titles and abstracts were screened against the inclusion criteria, resulting in 145 articles eligible for full-text analysis.
4. Inclusion: A final number of 105 studies were included in the narrative synthesis. These were selected based on mechanistic relevance, consistency of reported data, and direct applicability to dental restorative procedures.

Filters applied during the search included English language, Full-text availability, and Peer-reviewed articles. The primary focus was on articles published between 2021 and 2025. Foundational studies from 2015 to 2020 were included if they introduced key concepts still relevant today. Review articles were used for contextual discussion but not as primary evidence sources.

## 3. Antibacterial and Bioactive Nanomaterials for Restorative Dentistry

### 3.1. Context

The formation of bacterial biofilm at the interface between the restoration and the dental tissue is the leading cause of caries recurrence and composite restoration failure, along with other factors such as mechanical stress or poor marginal adaptation [32,33]. Even under optimal clinical conditions, microleakage promotes colonization by *Streptococcus mutans* and *Lactobacillus* spp., microorganisms involved in the initiation of secondary caries. Although *Streptococcus mutans* and *Lactobacillus* species have been linked to the onset and development of secondary caries, there is growing evidence to support the ecological plaque hypothesis, which holds that caries development is caused by dysbiotic changes within a complex polymicrobial biofilm rather than the dominance of a single pathogen. At restoration margins, acidogenic microbial consortia, which include non-mutans streptococci and other opportunistic species, work together to promote mineral breakdown, biofilm development, and local pH lowering. Therefore, rather than focusing just on specific bacterial species, antibacterial restorative techniques should be assessed in terms of their capacity to modify the behavior of polymicrobial biofilms and ecological equilibrium [34,35].

In this context, the integration of nanomaterials with antibacterial properties into composites, adhesives, and cements has become a central strategy in modern dental materials research [36,37,38].

### 3.2. Mechanisms of Action

The main mechanisms which scientists have documented include three methods: (1) the controlled release of metal ions (Ag^+^, Zn^2+^, Cu^2+^u^2+^) which penetrate bactel cell membranes, (2) the generation of reactive oxygen species (ROS) leading to oxidative stress–induced DNA damage, and (3) contact-killing mechanisms mediated by cationic particle surfaces [39,40,41]. Beyond these, two other relevant mechanisms are gaining attention: (4) the creation of anti-adhesive/protein-repellent surfaces, often using zwitterionic polymers like 2-methacryloyloxyethyl phosphorylcholine (MPC), which prevent initial bacterial attachment, and (5) pH-buffering or alkalizing effects, which counteract the acidic microenvironment produced by cariogenic bacteria, thus inhibiting their proliferation and promoting remineralization. It should be mentioned that the production of reactive oxygen species (ROS) and the release of ions are both strongly reliant on the environment and the materials used. Numerous contextual parameters, such as particle size, surface functionalization, nanomaterial composition, and surrounding oral circumstances, affect their intensity and biological significance. For instance, local pH variations, salivary composition, and protein adsorption at the material surface all influence the release of metal or remineralizing ions, whereas photocatalytic ROS generation by TiO_2_-based systems is highly sensitive to light exposure and wavelength. As a result, antibiotic efficacy measured in controlled laboratory settings may differ from that in therapeutically relevant settings, highlighting the importance of contextualizing mechanistic results.

The classification of nanomaterials used in restorative dental therapy, according to composition, mechanism of action, and clinical applications, is detailed in Table 1.

Experimental studies suggest that metal-based nanoparticles may inhibit secondary caries by exerting antibacterial activity through multiple mechanisms of action; however, most supporting evidence is currently derived from laboratory-based investigations [42,43,44,45].

Principal antibacterial mechanisms, advantages, and limitations of nanomaterials used in dental composites and adhesives are synthesized in Table 2.

### 3.3. Metallic Nanoparticle

Experimental studies indicate that incorporating metallic nanoparticles—particularly silver and zinc oxide—at low weight percentages (typically 0.5–1.0 wt%) can achieve significant biofilm reduction while preserving the mechanical integrity of resin-based composites and adhesives. However, optimal concentration thresholds vary with resin matrix composition, filler loading, and polymerization system, and therefore cannot be generalized across different restorative formulations.

Building on the general antibacterial mechanisms outlined above, scientists have conducted extensive research on silver nanoparticles (AgNPs), which are among the most-studied antibacterial compounds. The production of Ag^+^ ions disrupts bacterial respiratory enzymes, which eventually causes cell death [41,46,47,48]. The addition of AgNPs at low concentrations to composites and adhesives results in significant biofilm reduction while preserving their mechanical strength [43,49,50,51,52,53].

Zinc oxide nanoparticles (ZnO NPs) leverage their antimicrobial properties by releasing Zn^2+^ ions and generating reactive oxygen species (ROS), with several experimental studies indicating sustained antibacterial effects and acceptable biocompatibility [54].

Copper nanoparticles (CuNPs) offer a similar effect but are more economical; however, high concentrations can compromise the material’s chemical stability. Their antibacterial efficacy is recognized, but chemical instability in humid conditions is well documented [55,56].

Titanium dioxide (TiO_2_) exhibits photocatalytic activity under visible light when doped with Ag or Zn, producing free radicals that enhance its antibacterial activity beyond ultraviolet light [57,58]. When adding metallic nanoparticles to restorative materials, several clinically significant issues need to be considered from a translational standpoint. Changes in color and opacity caused by the presence of copper or silver nanoparticles may affect esthetic outcomes, especially in anterior restorations. Particle size, surface functionalization, and resin permeability also affect long-term ion-release kinetics, and antibacterial activity frequently declines as the ion reservoir depletes. Furthermore, under intraoral settings, interactions with salivary proteins and acquired pellicle formation can alter the surface availability of nanoparticles, perhaps reducing both ion release and contact-based antibacterial activities. These elements emphasize the importance of assessing metallic nanoparticle-based systems for their long-term aesthetic stability and functional endurance in clinically relevant settings, as well as their short-term antibacterial efficacy.

Research findings show that experimental composite systems exhibit greater stability and antibacterial performance when dopants are combined with sol–gel surface treatment methods [59,60]. However, the antibacterial efficacy reported for metallic nanoparticles is strongly influenced by particle size, concentration, surface functionalization, and exposure time. Consequently, direct comparison between studies is limited, and reproducibility across different experimental platforms remains a challenge.

### 3.4. Cationic Monomers and Natural Nanopolymers

The development of antibacterial materials requires researchers to focus on cationic monomers, such as dimethylaminohexadecyl methacrylate (DMAHDM), which kill bacteria by disrupting their membranes upon contact [61]. In vitro studies have reported substantial reductions in *Streptococcus mutans* biofilm viability following the incorporation of DMAHDM into resin-based materials, often exceeding 90% under controlled experimental conditions. However, these outcomes are highly model-dependent and influenced by factors such as biofilm maturity, exposure time, and testing configuration, and therefore should not be directly extrapolated to clinical performance [62]. The surface becomes more resistant to bacterial adhesion because DMAHDM combines with 2-methacryloyloxyethyl phosphorylcholine (MPC) to create a highly hydrophilic surface layer [63]. Chitosan-based nanocomposites combined with ZnO or Ag nanoparticles have demonstrated sustained antibacterial activity in laboratory-based aging models, typically over periods ranging from several days to a few weeks, depending on nanoparticle loading and storage conditions [22,64,65]. These effects have primarily been observed in controlled aqueous or simulated oral environments [66,67,68]. At the same time, evidence regarding long-term antibacterial persistence following prolonged aging or repeated exposure to salivary proteins remains limited. Consequently, although these systems show promise for extended antimicrobial performance, their durability under clinically relevant aging conditions remains to be validated.

### 3.5. Carbon-Based Nanomaterials

The combination of mechanical strength and antimicrobial properties makes graphene and graphene oxide (GO) substances widely used [69]. The bacterial cell wall undergoes mechanical failure, while ROS production serves as the primary mechanism for killing bacteria. The addition of GO to adhesives and glass-ionomer cements decreases biofilm density while increasing the dentin–adhesive interface’s resistance to damage [70]. Structural characteristics such as degree of oxidation and lateral sheet size have a significant impact on the antibacterial and physicochemical behavior of carbon-based nanomaterials, especially graphene oxide (GO). Although higher oxidation levels can change the mechanical reinforcement potential and decrease electrical conductivity, they can improve hydrophilicity and make dispersion easier in wet conditions. On the other hand, smaller flakes might show distinct cellular connections and biological reactions, whilst bigger lateral dimensions have been linked to increased mechanical membrane-disruptive effects. Uniform dispersion inside resin matrices continues to be a major difficulty from the standpoint of formulation. The mechanical characteristics, optical homogeneity, and antibacterial consistency of graphene-based nanomaterials may be compromised by their significant agglomeration tendency. Furthermore, adding GO to resin-based systems may have an impact on polymerization behavior and viscosity, which could have an impact on handling properties, degree of conversion, and monomer mobility. When converting carbon-based nanomaterials into restorative dental materials, careful optimization is necessary since these formulation-dependent effects are extremely sensitive to filler loading, surface functionalization, and mixing techniques.

Ongoing research continues to examine the long-term biocompatibility of carbon-based nanomaterials and the potential for adverse biological effects associated with their accumulation in oral tissues [71].

### 3.6. Integration into Composites and Adhesives

**Figure 1:**
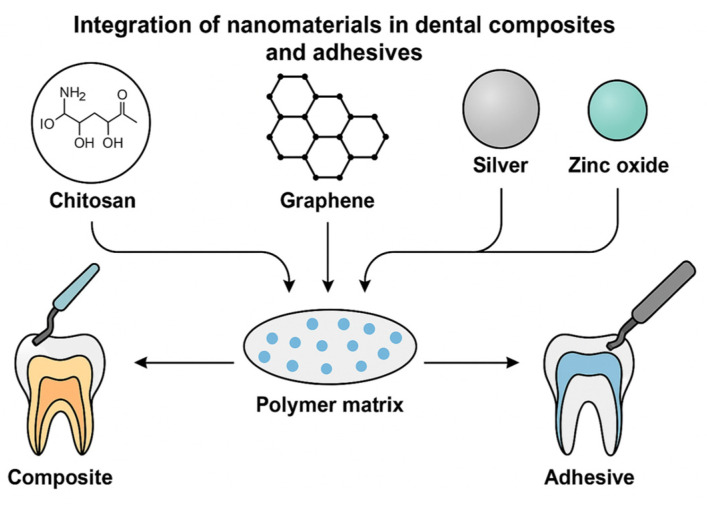
Nanomaterials function within dental composites and adhesives.

The performance of nanocomposite (Figure 1) materials depends on the compatibility between the inorganic and organic phases. The process of nanoparticle agglomeration compromises mechanical and optical properties, so researchers use sol–gel synthesis and silane functionalization to achieve better dispersion uniformity [72]. The development of pH-sensitive release systems that provide active antibacterial defense has become more prevalent in modern restorative materials [73].

From a forward-looking standpoint, new research has suggested that combining stimulus-responsive nanomaterials with artificial intelligence-assisted monitoring could maximize the efficacy of restorative materials [74]. However, there is currently no clinical validation or commercially accessible devices that provide real-time tracking of restorative behavior, making such techniques largely theoretical or limited to proof-of-concept studies. Therefore, rather than being clinically proven tactics, these ideas should be viewed as directions for exploratory studies.

### 3.7. Limitations and Future Directions

To improve comparability across studies and support clinical translation, future research on antibacterial and bioregenerative restorative nanomaterials would benefit from adopting standardized testing endpoints. Aging protocols should systematically include thermocycling, long-term water aging, and pH cycling to better simulate the dynamic oral environment and assess material stability over time. Antibacterial performance should be evaluated using both single-species and multispecies biofilm models, ideally incorporating salivary protein conditioning and acquired pellicle formation to reflect clinically relevant biofilm behavior. In parallel, cytotoxicity assessment should align with ISO 10993–1:2025 [75] relevant endpoints, including cell viability, inflammatory response, and dose-dependent effects under prolonged exposure. Importantly, antimicrobial efficacy should be re-evaluated after aging procedures to determine the durability of antibacterial function and the potential impact of ion depletion, surface modification, or protein adsorption. The use of such standardized endpoints would facilitate meaningful cross-study comparisons and accelerate the translation of experimental materials into clinically viable restorative solutions.

The materials show promising results in laboratory tests, yet multiple obstacles must be overcome before they can be considered reliable for medical applications. The production of nanomaterials faces three significant challenges: expensive manufacturing processes, possible accumulation of toxic substances, and insufficient worldwide regulations for measuring their performance.

Current research increasingly investigates hybrid nanomaterials that combine antibacterial activity with regenerative biofunctionality, while acknowledging the experimental nature of these approaches [76].

Additive manufacturing technologies and AI-assisted design platforms are being explored as potential tools for personalized restorative solutions, although their clinical implementation remains preliminary [77].

Overall, while nanomaterial-based strategies demonstrate substantial antibacterial and bioactive potential in experimental models, their reported performance should be interpreted cautiously, as outcomes are frequently model-dependent and influenced by experimental design parameters.

## 4. Bioregenerative Nanomaterials in Restorative Dental Therapy

### 4.1. Concept and Biological Basis

Restorative dentistry now focuses on bioactive and biologically interactive materials, moving beyond purely passive repair approaches as a result of advances in materials science [78]. The current objective extends beyond structural replacement to include the stabilization of the tooth–material interface and the support of natural biological processes, primarily remineralization and bioactivity, which may contribute to dentin repair and long-term tissue preservation [79]. Bioregenerative materials are substances that form durable chemical bonds with tooth structures while producing beneficial biological effects, primarily related to bioactivity and mineral-based repair at the tooth–material interface. The controlled release of Ca^2+^, PO_4_^3−^ and Si^4+^, ions becomes possible through particles with significant surface areas that exhibit nanoscale reactivity for apatite formation [78,79,80].

### 4.2. Nanohydroxyapatite (nHAp)

The bioactive material nanohydroxyapatite (nHAp) has been reported because its chemical structure matches the natural mineral components of dental enamel and dentin. The 20–80 nm range of particles sticks to dental surfaces, which have been demineralized to create conditions for calcium and phosphate ion accumulation, leading to ongoing remineralization. Research conducted in laboratory settings shows that the material strengthens tooth enamel through two mechanisms: increasing enamel hardness and forming a mineral-based bond between the material and the tooth structure. The biocompatibility of nHAp is demonstrated by positive results in tests with pulp cells and gingival fibroblasts. The substance finds clinical application through its addition to composites, cements, and remineralizing adhesives, which help decrease dentin hypersensitivity. The addition of fluoride or zinc ions to nHAp surfaces via surface functionalization methods produces materials that exhibit improved antimicrobial activity and enhanced tissue-regeneration capabilities [81].

Alternative natural sources of calcium phosphates have also been explored for dental applications. Hydroxyapatite derived from mollusk shells has been shown to exhibit favorable physicochemical properties, biocompatibility, and remineralization potential, supporting its use in restorative and preventive dentistry [82]. Although nHAp has been incorporated into various clinical products, including toothpastes, adhesives, and restorative materials, many reported benefits are primarily derived from in vitro microhardness measurements, surface remineralization, or laboratory-based studies. Evidence supporting long-term clinical outcomes, durability, and sustained performance under intraoral conditions remains comparatively limited [81].

### 4.3. Bioglass

The biological activation of Bioglass (SiO_2_–CaO–Na_2_O–P_2_O_5_) occurs through the formation of carbonated hydroxyapatite when it contacts saliva [18,83]. The nanoscale bioglass (nBG) releases its Ca^2+^ and Si^4+^ ions at a faster rate, which helps cells attach to bone tissue and promotes odontoblastic cell differentiation. The addition of nBG to cement and composite materials enhances chemical bonding to dental materials and simultaneously strengthens their ability to remineralize [84,85]. The addition of strontium, fluoride, and zinc ions to bioglass creates new antibacterial capabilities that also enhance apatite formation. Porous structures for pulp regeneration can be fabricated using sol–gel technologies and 3D printing methods [86]. In addition to its remineralizing potential, bioglass exhibits an alkalizing effect by releasing sodium, calcium, and silicate ions, which can locally increase pH and counteract the acidic conditions associated with cariogenic biofilms. This pH-buffering behavior is considered relevant to caries prevention, particularly at restoration margins where recurrent acid challenges occur.

However, it should also be noted that high bioglass loadings within resin-based composites or cements may compromise mechanical properties, such as flexural strength and wear resistance, depending on particle size, dispersion, and matrix compatibility. Consequently, optimization of bioglass content remains essential to balance bioactivity with structural performance.

### 4.4. Calcium Phosphates and Calcium Silicates

Calcium phosphate nanophases (ACP, TCP, NACP) and calcium silicates (CS, LS) have been reported in “in vitro” and preclinical models to support mineral repair processes and in the modulation of cellular responses at the dentin–pulp interface. NACP releases Ca^2+^ and PO_4_^3−^ ions under acidic conditions, ensuring adaptive remineralization [87]. The dental pulp stem cells differentiate in response to calcium silicate compounds, which also stimulate the formation of reparative dentin [88,89]. The combination of particles with natural biopolymers such as collagen, gelatin, and chitosan results in bioactive scaffolds that serve as regenerative therapy tools [90]. Bioregenerative effects of major nanomaterials used in restorative dentistry are synthesized in Table 3.

### 4.5. Hybrid Nanocomposites

Scientists have developed hybrid nanocomposites by adding nanoparticles to polymeric matrices, which provide both enhanced mechanical strength and precise delivery of bioactive substances. The representative systems include nHAp–chitosan, NACP–DMAHDM, and bioglass–silicate composites. These compounds have two functions: restoring minerals to the sticky surface and preventing biofilm development. Additionally, it has been shown that functionalized nanocellulose, when augmented with Ca^2+^ and Si^4+^, can exhibit self-healing properties in adhesive compositions [91,92,93]. From a practical formulation perspective, the incorporation of hybrid nanocomposites into resin-based systems may also influence polymerization kinetics and the degree of conversion. High nanofiller loadings, increased viscosity, or light-scattering effects can reduce monomer mobility and light penetration, potentially affecting curing efficiency and final material performance. These factors represent significant constraints that must be carefully optimized to ensure adequate polymerization and long-term clinical reliability.

### 4.6. Regenerative Mechanisms and Cellular Effects

In addition to mineral-related effects, some bioactive nanomaterials have been reported to modulate inflammatory responses in experimental models. In particular, in vitro cell culture studies and selected preclinical investigations have described associations with reduced expression of pro-inflammatory markers, such as TNF-α and IL-6, under controlled conditions. However, these effects are highly context-dependent, varying with material composition, concentration, exposure time, and model system, and should not be extrapolated directly to clinical inflammatory outcomes [94].

### 4.7. Limitations and Future Directions

The development of antibacterial and bioregenerative nanomaterials continues to face several significant challenges, including the control of ion release, the maintenance of chemical stability between organic and inorganic components, and the limited availability of long-term clinical data. At the experimental level, pH-responsive systems capable of triggering ion release under acidic conditions have been successfully demonstrated in vitro and in preclinical models, primarily using laboratory-based demineralization and biofilm simulations. These approaches have shown the feasibility of adaptive, environment-sensitive behavior under controlled conditions. In contrast, the integration of smart nanomaterials with growth factors or AI-driven predictive modeling remains mainly conceptual. While preliminary computational and proof-of-concept studies suggest potential benefits for personalized and biologically adaptive restorative treatments, such strategies have not yet been validated in clinically relevant models. Consequently, these concepts should be regarded as promising future research directions rather than established therapeutic solutions.

## 5. Dual-Function Nanomaterials with Antibacterial and Bioregenerative Activity

### 5.1. Concept and Rationale

The concept of dual-function nanomaterials defines an emerging category of materials that combine antibacterial and regenerative properties, providing protection against infections and stimulating tissue repair [21]. These materials address the limitations of traditional strategies: metal nanoparticles can be cytotoxic at high doses [95], and bioactive materials, although they promote remineralization, do not prevent microbial colonization. By integrating both functionalities into a single system, a biological synergy is achieved, in which the elimination of bacteria and the stimulation of regeneration are complementary processes [21]. Dual-function nanomaterials provide potential benefits, but their design entails significant trade-offs that need to be carefully considered. Particularly at higher concentrations, antimicrobial additions such as metal nanoparticles and cationic compounds may disrupt resin polymerization by altering light transmission, radical propagation, or monomer mobility. In a similar vein, when added at high loadings, ion-releasing fillers that promote bioactivity and remineralization may weaken the material’s strength, resistance to wear, or stability over time. In order to ensure adequate antibacterial and regenerative benefits without compromising polymerization efficiency or mechanical reliability, dual-function systems’ clinical effectiveness thus relies on striking a careful balance between biological activity and material durability.

### 5.2. Synergistic Action Mechanisms

**Figure 2:**
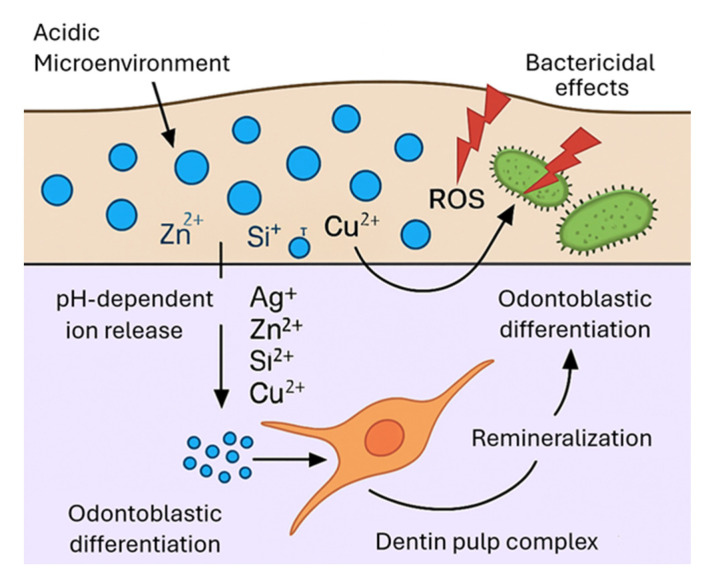
Schematic representation of dual antibacterial and bioregenerative mechanisms of dental nanomaterials.

Dual-function nanomaterials combine antibacterial and bioregenerative effects through distinct but complementary mechanisms (Figure 2). Antimicrobial activity may arise either from contact-killing systems, such as cationic surfaces that disrupt bacterial membranes upon direct contact, or from release-based mechanisms, in which metal-derived ions (e.g., Ag^+^, Zn^2+^, Cu^2+^) induce oxidative stress, membrane damage, and metabolic disruption in microbial cells. These approaches differ in their dependence on ion availability, surface accessibility, and environmental conditions. In parallel, bioactive ions such as Ca^2+^, PO_4_^3−^, and Si^4+^ contribute to apatite formation and are associated with odontoblastic cell responses under experimental conditions [22]. Importantly, the most effective dual-function systems are not defined solely by strong acute antibacterial effects, but by their ability to maintain durable antimicrobial performance after aging, while preserving bioactivity and mechanical integrity. Achieving this balance remains a central design challenge in the development of clinically relevant dual-function restorative materials.

### 5.3. NACP–DMAHDM Composites

One of the most representative dual systems is the composite based on nano-calcium phosphate (NACP) and dimethylaminohexadecyl methacrylate (DMAHDM). NACP provides remineralizing ions and a pH-responsive effect in acidic conditions, while DMAHDM exerts antibacterial action through contact killing. In vitro studies show a reduction in the viability of *S. mutans* biofilm and a tendency to restore local pH. Integration into adhesives/cements provides double protection without altering the mechanical properties reported for similar systems [96]. From a durability perspective, the long-term effectiveness of contact-killing mechanisms in NACP–DMAHDM composites may be influenced by protein-rich oral environments. Adsorption of salivary proteins and acquired pellicle formation can partially mask cationic active sites, potentially reducing antibacterial efficiency over time. In addition, several experimental studies have evaluated the performance of NACP–DMAHDM systems after thermocycling and prolonged water aging, reporting that while antibacterial activity is often retained, its magnitude may decrease depending on aging duration, material formulation, and surface accessibility. These findings highlight the importance of assessing both antibacterial persistence and bioactivity following clinically relevant aging protocols.

### 5.4. Hybrid Systems of Ag–nHAp and ZnO–Bioglass

The antibacterial properties of silver and the bioactivity of hydroxyapatite are combined in hybrid Ag–nHAp composites, which prevent biofilm formation and support enamel remineralization. The ZnO–bioglass composites serve as two-in-one materials because they deliver Zn^2+^ and Si^4+^ ions at particular times to control bacterial growth and enhance apatite formation.

Depending on the material formulation and testing paradigm, hybrid systems that combine Ag–nHAp or ZnO–bioglass have shown satisfactory biocompatibility in experimental investigations when added at low weight percentages, usually 0.5–1.0 wt%. Under carefully monitored in vitro conditions, antibacterial activity can be achieved at these concentrations while preserving cytocompatibility with cells derived from tooth pulp. However, esthetic issues also need to be taken into account, especially for systems that incorporate ser. The use of Ag nanoparticles in restorative areas that require esthetics may be limited by the potential for discoloration or opacity changes, particularly at higher loadings. To balance antibacterial efficacy, biocompatibility, and aesthetic results, silver content optimization remains crucial [97,98].

### 5.5. Chitosan- and Graphene-Based Nanocomposites

In vitro cell culture systems and selected preclinical models have shown that chitosan–graphene oxide-based nanocomposites may enhance odontogenic marker expression, including DSPP and DMP-1. These findings imply odontogenic responses under controlled laboratory settings, while in vivo organized reparative dentin production is lacking. Thus, such studies suggest bioactive potential rather than restorative effects [99,100].

### 5.6. Smart Nanomaterials with Controlled Release

Systems based on biodegradable micro- and nanocapsules (e.g., PLGA, PCL) and pH-sensitive hydrogels are being actively explored for their ability to release bioactive ions and/or antimicrobial agents in a controlled manner under acidic conditions. When integrated into adhesives or composite materials, such pH-responsive approaches aim to reduce secondary caries risk by enabling adaptive ion release and maintaining a microenvironment favorable to remineralization. The broader concept of smart restorations, including monitoring or optimization via artificial intelligence and digital workflows, should currently be regarded as an emerging, largely conceptual research direction. While preliminary computational models and proof-of-concept studies suggest potential benefits, AI-assisted optimization of restorative behavior has not yet been validated in clinically established workflows and remains the subject of ongoing investigation [101].

### 5.7. Cellular Compatibility and Biointegration

Medical research needs to advance into clinical practice through a process which depends on biocompatibility as its fundamental requirement. Cell protection from toxic damage is possible through the use of small amounts of metal nanoparticles and methods that stabilize bioactive matrices. The Ag–nHAp and ZnO–bioglass systems exhibit greater expression of odontogenic markers during laboratory tests and do not harm dental pulp stem cells. The combination of chitosan with graphene/GO networks enables cell attachment and differentiation, thereby improving material-tissue bonding. From a translational standpoint, evaluation of cellular compatibility and biointegration of restorative nanomaterials should be guided by a clear set of standardized biological endpoints. These include cytotoxicity and genotoxicity assessments in accordance with ISO 10993 standards, evaluation of inflammatory responses (e.g., cytokine expression, macrophage activation), and quantification of ion release profiles to ensure exposure remains within biologically acceptable limits. For systems containing metallic nanoparticles, additional consideration of tissue distribution, accumulation, and clearance is warranted, particularly under long-term exposure scenarios. Systematic assessment of these parameters is essential to ensure biological safety and to support meaningful comparison across studies.

### 5.8. Clinical and Technological Perspectives

Advances in nanomanufacturing, 3D printing, and artificial intelligence support the design of multifunctional materials with predictable performance.

Computer modeling and machine learning algorithms can correlate composition with biological properties, reducing testing costs through data-driven material design.

From a clinical perspective, these materials promise to reduce secondary caries, accelerate pulp healing, and extend the lifespan of restorations; however, standardization of testing, ecotoxicological evaluation, and regulatory acceptance remain essential for their translation into everyday practice. From a translational standpoint, progress toward clinical implementation of antibacterial and bioregenerative nanomaterials requires alignment with clinically relevant testing paradigms and regulatory expectations. In particular, material performance should be evaluated under combined aging and biofilm challenge models, incorporating thermocycling, long-term water or pH aging, and exposure to multispecies biofilms with salivary pellicle formation, to better simulate intraoral conditions.

In parallel, regulatory translation necessitates systematic assessment of ion release profiles, leachable components, and potential nanoparticle migration, especially for metal-containing or degradable systems. Quantification of release kinetics, dose-dependent exposure, and long-term stability is essential to support safety evaluation and compliance with emerging regulatory frameworks. Anchoring technological innovation to these clinically and regulatory-relevant endpoints will be critical for advancing laboratory concepts toward routine restorative practice.

## 6. Technological Advances and Future Directions

The development of nanotechnology is ushering in a new era for dental restorative materials, enabling the creation of bioactive systems that function as personalized medical solutions. This progress is driven by key technological advancements that allow for the precise design and fabrication of materials with tailored biological and mechanical properties.

### 6.1. Smart and Responsive Materials

Smart materials that respond to environmental stimuli such as pH, temperature, or the presence of bacteria represent a significant leap forward. These systems can, for instance, release remineralizing ions only in acidic conditions (pH-responsive) or trigger antibacterial photocatalytic reactions when exposed to blue light. Nanocomposites based on PLGA microcapsules are being explored for the controlled release of antimicrobial and mineral agents, offering an adaptive defense mechanism. Furthermore, self-healing materials containing microencapsulated monomers can repair microcracks, extending the functional lifespan of restorations and marking a transition from passive to dynamic, self-regulating systems.

### 6.2. Bioactive and Regenerative Nanomaterials

The core of modern restorative materials lies in their bioactivity. The ability to control material structure at the nanoscale through advanced synthesis methods—such as sol–gel, hydrothermal processing, and microemulsion—allows for the production of homogeneous nanoparticles with optimized crystallinity and shape. Surface functionalization with coupling agents (e.g., silanes, bioactive peptides) enhances the bond between the inorganic fillers and the polymer matrix. Moreover, doping with bioactive ions (e.g., Sr^2+^, Zn^2+^, Mg^2+^) can confer multiple benefits, including osteogenic stimulation, enhanced antimicrobial activity, and promotion of angiogenesis, allowing for the creation of materials tailored to specific biological requirements.

### 6.3. Digital and Additive Manufacturing

Additive manufacturing, particularly 3D printing, allows for the fabrication of complex, patient-specific scaffolds and restorative frameworks. By incorporating nanofillers into 3D-printable resins, it is possible to create structures with enhanced mechanical properties and controlled ion release profiles. This technology enables the design of personalized regenerative templates that meet both the structural and functional needs of the patient, paving the way for truly customized dental restorations.

### 6.4. AI Integration

The integration of artificial intelligence (AI) and machine learning (ML) is revolutionizing the design and optimization of dental nanomaterials. AI algorithms can analyze vast datasets to correlate material composition, structure, and synthesis parameters with their resulting mechanical and biological performance. This predictive capability accelerates the research and development cycle and reduces validation costs. When combined with 3D digital modeling of the oral cavity, AI can simulate material behavior under clinically relevant conditions, transforming restorative dentistry into a predictive and adaptive system where the material, the biological environment, and the clinician are interconnected [29].

### 6.5. Sustainability and Regulatory Considerations

Despite technological progress, the clinical translation of nanomaterials raises important questions regarding long-term safety and environmental impact. The potential for nanoparticles to cross biological barriers and accumulate in tissues necessitates thorough assessments of their biodistribution and ecotoxicology. Regulatory bodies like the ISO (with standards such as ISO 10993 for biocompatibility) and the FDA are establishing frameworks for the characterization and testing of medical nanomaterials. Concurrently, there is a growing focus on developing biodegradable and sustainable materials, such as nanocomposites based on natural polymers (e.g., chitosan, curcumin) or eco-friendly bioceramics, as an ethical and scientific priority [102,103].

### 6.6. Future Directions

Antibacterial and bioregenerative nanoparticles for restorative dentistry should focus on the following directions:

- To ensure repeatability and cross-study comparability, standardized experimental methods were developed to assess antibacterial activity, bioactivity, and mechanical performance.
- To evaluate long-term material stability using therapeutically relevant aging methods, such as thermocycling, prolonged water storage, and pH cycling, to determine the durability of antibacterial and bioactive functions.
- To implement sophisticated biofilm models with salivary pellicle development to better imitate intraoral microbial habitats.
- To support the integration of clinically significant objectives, such as secondary caries prevention, marginal integrity, restorative longevity, and biological safety.

Focused research is needed to reconcile laboratory-based innovation with predictable clinical performance.

## 7. Conclusions

Nanotechnology has transformed restorative dentistry from a discipline of mechanical reconstruction into one of assisted biological healing.

Current Status of Evidence (In Vitro and Preclinical): Experimental evidence confirms the efficacy of metallic nanoparticles (AgNPs, ZnO), cationic monomers (DMAHDM), and natural nanopolymers in inhibiting biofilm formation without compromising the mechanical properties of the materials. Simultaneously, systems based on nanohydroxyapatite (nHAp), bioglass, and amorphous calcium phosphates (NACP) promote active remineralization and maintain pulp vitality. Dual-function materials, such as the NACP–DMAHDM system, represent the current state-of-the-art, offering an adaptive response to fluctuations in the oral pH.

Limitations and Uncertainties: Despite laboratory success, clinical adoption is hindered by a significant translational gap. There is a lack of long-term data on durability after aging in the oral environment, the safety of metallic nanoparticle biodistribution, and the ecotoxicological impact. Most studies remain limited to monoculture models (e.g., *S. mutans*), ignoring the complexity of multispecies biofilms and their interaction with the salivary pellicle.

Future Perspectives and Needs: The transition to a predictive restorative dentistry depends on:

- Standardization: Implementation of rigorous testing protocols (e.g., ISO 10993) that include thermal and pH cycling;
- Technology: Use of AI for formulation optimization and 3D printing for personalized regenerative structures;
- Sustainability: Development of bio-fabricated materials based on natural compounds (e.g., nano-curcumin, chitosan) to minimize cytotoxicity risks;

In conclusion, while the potential of nanomaterials is vast, their clinical validation depends on the generation of robust evidence confirming their long-term biocompatibility and functional performance.

## Figures and Tables

**Table 1:** Classification of biofunctional nano-enabled components and materials in restorative dentistry.

Main Category	Representative Examples	Primary Mechanism of Action	Clinical Applications and Relevant Effects	Key Limitations
Antibacterial Nanomaterials	AgNPs, ZnO, CuO, TiO_2_, Cationic monomers (DMAHDM), Chitosan, Graphene oxide	- Controlled release of metal ions (Ag^+^, Zn^2+^, Cu^2+^) - Generation of reactive oxygen species (ROS) - Contact-killing through electrostatic interactions - Inhibition of bacterial adhesion and biofilm formation	- Reduction in marginal secondary caries - Prevention of secondary inflammation - Incorporation into composites, cements, adhesives, and sealants	- Potential cytotoxicity at high concentrations - Discoloration (especially AgNPs) - Interference with polymerization - Long-term ion release stability
Bioregenerative Nanomaterials	Nanohydroxyapatite (nHAp), Bioglass (BG), Amorphous Calcium Phosphate (ACP, NACP), Calcium Silicates	- Release of Ca^2+^, PO_4_^3−^ and Si^4+^ ions - Promotion of secondary apatite nucleation - Stimulation of odontoblastic differentiation - Local pH buffering and rebalancing	- Remineralization of enamel and dentin - Support for dentin–pulp regeneration - Reduction in dentin hypersensitivity - Improved bonding at the dentin–adhesive interface	- Mechanical properties can be compromised at high filler loads - Release kinetics are difficult to control - Efficacy is highly dependent on the oral environment (pH, saliva)
Dual-Function Nanomaterials	NACP–DMAHDM, Ag–nHAp, ZnO–bioglass, Chitosan–GO, Zn- or F-doped bioglass	- Synergistic release of antibacterial and remineralizing ions- pH-responsive behavior (acid-triggered ion release) - Combined contact-killing effect and apatite formation	- “Smart” restorations with biologically adaptive behavior - Prevention of secondary caries and enhancement of tissue healing - Use in bioactive composites, cements, and base materials	- Complex formulation challenges - Balancing antimicrobial efficacy vs. cytotoxicity - Long-term durability and safety not yet established in clinical settings

**Table 2:** Antibacterial Mechanisms and Clinical Implications of Major Nanomaterials Used in Restorative Dental Therapy.

Nanomaterial	Primary Antibacterial Mechanism	Major Advantages	Limitations/Challenges
Silver nanoparticles (AgNPs)	Release of Ag^+^ ions → denaturation of enzymatic proteins and disruption of the bacterial membrane; generation of reactive oxygen species (ROS)	Broad-spectrum antibacterial activity; rapid and persistent effect; good compatibility with resin composites	Potential cytotoxicity at higher concentrations; gray discoloration; incomplete understanding of biodistribution
Zinc oxide nanoparticles (ZnO NPs)	Release of Zn^2+^ and ROS generation → oxidative stress and inhibition of bacterial metabolism	Better biocompatibility than AgNPs; high chemical stability; synergistic effects with other agents	pH- and concentration-dependent antibacterial activity; potential interference with resin polymerization at higher loadings; variability in long-term ion release under oral conditions
Copper nanoparticles (CuNPs)	Release of Cu^2+^ → disruption of bacterial cell walls and inhibition of microbial DNA	Low cost; antibacterial and antifungal efficacy; good stability within composite matrices	Susceptible to oxidation and instability in moist environments; potential discoloration of the material
Titanium dioxide nanoparticles (TiO_2_ NPs)	Photocatalytic activation under UV or blue light → formation of bactericidal free radicals	Tunable photocatalytic properties; high stability; non-toxic in the absence of light	Require light activation; limited activity without irradiation; possible unwanted oxidative reactions
Cationic monomer DMAHDM	Contact killing—positively charged surfaces attract and disrupt bacterial membranes	Compatible with resin composites; long-lasting antibacterial effect; does not leach from the matrix	Active only upon direct contact; reduced efficiency in the presence of mature biofilm Contact-dependent antibacterial activity; reduced effectiveness against mature or thick biofilms; potential masking of cationic sites by salivary proteins and acquired pellicle formation
Chitosan (natural nanopolymer)	Electrostatic interaction with bacterial membranes → increased permeability and DNA inhibition	Excellent biocompatibility; biodegradable; additional anti-inflammatory properties	Low solubility at neutral pH; limited activity against Gram-negative bacteria
Graphene oxide (GO)	Mechanical membrane disruption (“cutting” effect) + ROS generation	Superior mechanical properties; synergistic effects with other agents; antibacterial activity without requiring metal ions	Potential pro-oxidative effects at high concentrations; biocompatibility still under investigation

**Table 3:** Bioregenerative Nanomaterials and Their Biological Effects on Dental Structures.

Material	Composition	Mechanism of Action	Biological Effects	Primary Evidence Level
nHAp	Ca_10_(PO_4_)_6_(OH)_2_	- Biomimetic nucleation - Ion release (Ca^2+^, PO_4_^3−^)	- Enamel/dentin remineralization - Occlusion of dentinal tubules - Stimulation of odontogenic differentiation	In vitro, Animal model
Bioglass	SiO_2_–CaO–Na_2_O–P_2_O_5_	- Apatite layer formation - Ion release (Ca^2+^, Si^4+^) - pH increase	- Dentin remineralization - Odontoblastic differentiation - Angiogenic potential	In vitro, Animal model
NACP	Amorphous Calcium Phosphate	- pH-responsive ion release - High surface reactivity	- Sustained remineralization - pH buffering capacity	In vitro
Calcium Silicates	CaSiO_3_, Ca_3_SiO_5_	- Hydration reaction - Ca(OH)_2_ formation - Ion release	- Dentin bridge formation - Pulp capping applications - Antibacterial effect (high pH)	In vitro, Clinical trial (limited)

## Data Availability

No new data were created or analyzed in this study. Data sharing is not applicable to this article.

## Abbreviations

The following abbreviations are used in this manuscript:

ACP	Amorphous Calcium Phosphate
AgNPs	Silver Nanoparticles
AI	Artificial Intelligence
BAG	Bioactive Glass
BG	Bioglass
CS	Calcium Silicates
CuNPs	Copper Nanoparticles
DMAHDM	Dimethylaminohexadecyl Methacrylate
DMP-1	Dentin Matrix Protein 1
DSPP	Dentin Sialophosphoprotein
FDA	Food and Drug Administration
FDI	World Dental Federation
GO	Graphene Oxide
ISO	International Organization for Standardization
LS	Lithium Silicates
ML	Machine Learning
MPC	2-Methacryloyloxyethyl Phosphorylcholine
NACP	Nano-Calcium Phosphate
nBG	Nanoscale Bioglass
nHAp	Nanohydroxyapatite
NCD	Non-Communicable Disease
PLGA	Poly(lactic-co-glycolic acid)
PCL	Polycaprolactone
QAM	Quaternary Ammonium Monomers
ROS	Reactive Oxygen Species
UV	Ultraviolet
ZnO NPs	Zinc Oxide Nanoparticles
